# Investigation of thrombin generation assay to predict vaso-occlusive crisis in adulthood with sickle cell disease

**DOI:** 10.3389/fcvm.2022.883812

**Published:** 2022-10-05

**Authors:** Guillaume Feugray, Fiston Kasonga, Maximilien Grall, Cécile Dumesnil, Ygal Benhamou, Valery Brunel, Véronique Le Cam Duchez, Agnès Lahary, Paul Billoir

**Affiliations:** ^1^Vascular Hemostasis Unit, CHU Rouen, Normandie Université, UNIROUEN, INSERM U1096, Rouen, France; ^2^Vascular Hemostasis Unit, CHU Rouen, Rouen, France; ^3^Department of Internal Medicine, CHU Rouen, Rouen, France; ^4^Department of Pediatric Onco-Hematology, CHU Rouen, Rouen, France; ^5^Department of Internal Medicine, CHU Rouen, Normandie Université, UNIROUEN, INSERM U1096, Rouen, France; ^6^Department of General Biochemistry, CHU Rouen, Rouen, France; ^7^Hematology Laboratory, CHU Rouen, Rouen, France

**Keywords:** thrombin generation assay, hypercoagulability, hemoglobinopathy, sickle cell disease, vaso-occlusive crisis

## Abstract

**Introduction:**

Sickle cell disease (SCD) is an inherited hemoglobinopathy disorder. The main consequence is synthesis of hemoglobin S leading to chronic hemolysis associated with morbidity. The aim of this study was to investigate Thrombin Generation Assay (TGA) to assess hypercoagulability in SCD and TGA parameters as biomarkers of vaso-occlusive crisis (VOC) risk and hospitalization within 1 year.

**Materials and methods:**

We performed TGA in platelet poor plasma (PPP) with 1 pM of tissue factor and 4 μM of phospholipid-standardized concentration, in duplicate for patients and controls. We measured thrombomodulin (TM), soluble endothelial Protein C Receptor and Tissue Factor Pathway Inhibitor (TFPI).

**Results:**

A total of 113 adult patients with SCD, 83 at steady state and 30 during VOC, and 25 healthy controls matched on age and gender were included. Among the 83 patients at steady state, (36 S/S-1 S/β^0^, 20 S/Sα^3^.^7^, and 19 S/C-7 S/β^+^) 28 developed a VOC within 1 year (median: 4 months [2.25–6]). We observed an increase of peak and velocity associated with a shortening of lagtime and time to peak (TTP) and no difference of endogenous thrombin potential (ETP) in patients compared to controls. TFPI (*p* < 0.001) and TM (*p* = 0.006) were significantly decreased. TGA confirmed hypercoagulability in all SCD genotypes and clinical status. The association of ETP > 1,207 nM.min and peak >228.5 nM presented a sensitivity of 73.5% and a specificity of 93.9% to predict VOC development within 1 year.

**Conclusion:**

We have demonstrated a hypercoagulable state in SCD associated with chronic hemolysis. These preliminary findings suggest that TGA parameters, as ETP and peak, could be used to predict VOC development within 1 year.

## Introduction

Sickle cell disease (SCD) is an inherited hemoglobinopathy disorder caused by mutations in *HBB* gene with amino-acid substitution on β globin chain, leading to the production of the abnormal hemoglobin S (HbS). HbS polymerization in deoxygenated condition induces chronic hemolytic anemia and vaso-occlusive crisis (VOC), associating frequent hospitalization, morbidity and mortality caused by organ failure like stroke, acute chest syndrome (ACS), osteonecrosis, leg ulcers, retinopathy, pulmonary hypertension, priapism, and nephropathy (1). Moreover, a hypercoagulable state is reported in SCD with increased venous thromboembolism (VTE) or pulmonary embolism (PE) (1–3).

The pathophysiology of SCD is described as acquired thrombophilia with a complex mechanism (4). Hemostatic abnormalities as elevated prothrombin 1 + 2 fragment, D-dimer, factor VIII (FVIII), von Willebrand factor (vWf), tissue factor (TF) and decreased natural anticoagulants (protein C and S) or ADAMTS13 activity were reported (1, 2, 4–7). However, few studies have evaluated tissue factor pathway inhibitor (TFPI) and thrombomodulin (TM) in SCD. Other processes are involved in this hypercoagulable state, in particular, exposure to phosphatidylserine microvesicles from erythrocytes, monocytes and platelets (8–10), and formation of neutrophil extracellular traps (NETs) (11). This hypercoagulable state amplifies red blood cell (RBC) aggregation/adhesion (12), oxidative stress, inflammation and endothelial injury. A hypothesis of VOC amplification is thromboinflammation, induced by hemolysis and RBC adhesion (1, 5, 13, 14).

Thrombin generation assay (TGA) has demonstrated its relevance in thrombotic risk assessment in hereditary or acquired thrombophilia (15–18). TGA is a global assay which allows to differentiate hypo- or hyper-coagulable profiles (19, 20). Several studies have been published on TGA *ex vivo* in patients with SCD in steady state but the results are inconsistent requiring further studies (21–23).

The aim of this study was to evaluate the use of TGA to assess hypercoagulable state in SCD, and to investigate whether TGA parameters could be used to predict VOC and hospitalization within a year.

## Materials and methods

### Study design, patients, and controls

All patients in the study were diagnosed and treated for SCD at Rouen University Hospital between September 2018 and June 2021. Patients were included during an annual visit in our tertiary center (24). Patients with VOC were included less than 24 h after admission to emergency department. All patients treated with hydroxyurea had been treated for at least 3 years. All patients received a systematic annual visit to determine VOC development within a year. Patients were analyzed in four subgroups based on genotype and clinical status:

• Homozygous SCD (S/S) or β^0^ thalassemia (S/β^0^) at steady state;
• Homozygous SCD with α^3^.^7^ thalassemia (S/Sα^3^.^7^) at steady state;
• Heterozygous SCD with C hemoglobin (S/C) or β^+^ thalassemia (S/β^+^) at steady state;
• Patients hospitalized for VOC with any genotype.

Prospective data were collected and completed from medical records. Clinical data included age, sex, and history of thrombosis. Patients on long-term anticoagulant therapy, pregnant women, patients aged less than 18 years, and patients with confirmed coagulation factor deficiency were excluded.

Blood samples were obtained from 25 healthy controls matched on age and gender, who had no history of bleeding, no thrombosis, no factor deficiency (evaluated with coagulation factor measurement), and no inherited thrombophilia (Factor V Leiden mutation, Factor II Leiden mutation, antithrombin, protein S and protein C deficiency). Informed consent was obtained from all subjects.

The study was performed in accordance with the Declaration of Helsinki on biomedical research involving human subjects. The study was approved by the institutional review board (Rouen University Hospital Authorization protocol number: E2021-78) and is declared in clinical trials (clinical trials registration number: NCT05376046).

### Sample collection

Platelet poor plasma (PPP) samples were obtained from the initial blood test, taken by antecubital venipuncture with a 21-gauge needle and collected in vacutainer tubes containing buffered 0.109 M trisodium citrate (Greiner) (1 part of citrate 3.2%/nine parts of blood). PPP was prepared 1 h after sampling, by double centrifugation of citrated blood for 15 min at 2,250 g. PPP was stored in aliquots at −80°C until analysis and run within 4 months with prior thawing in water at 37°C for 5 min.

Standard follow-up included dipotassium EDTA tubes (BD Vacutainer EDTA, Plymouth) for blood counts and plasma from lithium heparin tubes with gel separator (BD Vacutainer LH, Plymouth) for biochemical parameters.

### Standard coagulation assays

Prothrombin time (PT) (Neoplastin, Diagnostica Stago, Asnières sur Seine, France) and activated partial thromboplastin time (aPTT) (PTT-A, Diagnostic Stago, Asnières sur Seine, France) were measured with STAR Max. Fibrinogen levels were measured in plasma by Clauss clotting method (STA-Liquid Fib, Diagnostica Stago, Asnières, France). All hemostasis tests were performed in citrated plasma samples.

### Soluble thrombomodulin, soluble endothelial protein C receptor, and tissue factor pathway inhibitor

Human BDCA-3 (TM) (Quantikine^®^, Bio-Techne Brands, Abingdon, United Kingdom) and human soluble Endothelial Protein C Receptor (sEPCR) (DuoSet ELISA^®^, Bio-Techne Brands, Abingdon, United Kingdom) (FilterMax F3^®^, Molecular Devices, San Jose, CA, United States) were measured at 450 nm. TFPI was measured by immuno-enzymatic kit (Asserachrom^®^ Total TFPI, Diagnostica Stago, Asnières, France).

### Thrombin generation assay

Thrombin generation assay was performed in PPP in duplicate with 1 pM of tissue factor and 4 μM of phospholipid (PPP low reagent, Diagnostica Stago, Asnières, France). TGA was measured by Calibrated Automated Thrombography (CAT), Fluoroscan Ascent Fluorometer (Thermo Scientific Lab Systems^®^, Helsinki, Finland) and Thrombinoscope^®^ software (Thrombinoscope 5.0 BV^®^, Maastricht, Netherlands). We used 96-well plates (Immunlon^®^2HB, Thermo Scientific, Rochester, NY, USA). The TGA parameters of thrombin generation curve were considered: lagtime (corresponding to the first trace of thrombin formation), time to peak (TTP; time necessary for thrombin maximal value), peak (maximal thrombin concentration), endogenous thrombin potential (ETP; area under the thrombin time concentration curve), and velocity [calculated: peak/(TTP-lagtime)]. TGA was carried out according to International Society of Thrombosis and Hemostasis (ISTH) recommendations (25). The coefficients of variation (CV) for intra-assay and inter-assay were calculated with pooled normalized plasma. CV for intra-assay and inter-assay were, respectively, 1.7 and 9.2% for lagtime, 1.7 and 8.6% for TTP, 2.5 and 6.8% for ETP, 4.7 and 6.8% for peak, 9.4 and 8.8% for velocity. Experiments were done in duplicate for controls and patients. No result could be validated if the agreement between two wells had a variation of >10%.

### Hemolysis parameters

Hemoglobin, reticulocyte count (RET) and plasma lactate dehydrogenase levels (LDH) were measured in samples collected for routine follow-up, in parallel with those collected for thrombin generation. Blood counts were measured on XN-9000 (Sysmex, Villepinte, France). LDH and indirect bilirubin levels were determined on cobas^®^ 8000 chemistry analyzer (Roche Diagnostics, Mannheim, Germany).

### Other laboratory parameters

Hemoglobin profile was determined by high performance liquid chromatography (HPLC) (Variant II Biorad, CA, United States), by capillary electrophoresis on Capillarys 3 Octa^®^ (Kit hydragel hémoglobineSebia, Lisses, France) and iso-electrofocalisation. The presence of α^3^.^7^ thalassemia was determined using a single-tube, multiplex-PCR assay.

### Statistical analysis

Data are expressed as medians and interquartile ranges (IQR). Statistical analyses were performed with GraphPad Prism for Windows, version 9.2 (GraphPad Software, San Diego, CA, United States). Pearson’s correlation was used to determine the correlation between two variables. TGA parameters between patients and controls were compared using a Kruskall–Wallis ANOVA with Dunn’s multiple comparisons post-test or Mann–Whitney test. Receiver operating characteristic (ROC) curves were built for significant clinical characteristics. *P*-values < 0.05 were considered to be statistically significant.

## Results

### Demographic characteristics

A total of 113 patients with SCD were included in this study, 83 at steady state and 30 during VOC. Among the 83 patients at steady state 37 were S/S-S/β^0^, 20 were S/Sα^3^.^7^, and 26 were S/C-S/β^+^. Patient characteristics are presented in Table 1. Among the 83 patients at steady state, 28 developed a VOC within 1 year (median: 4 months [2.25–6.0]). Seventy-three were treated with hydroxyurea.

**TABLE 1:** Characteristics of study population.

	Controls (*n* = 25)	S/S-Sβ ^0^ (*n* = 37)	S/Sα ^3.7^ (*n* = 20)	S/C-Sβ ^+^ (*n* = 26)	VOC (*n* = 30)
**Clinical characteristics**					
Age (years)	38.2 ± 11.8	31.1 ± 11.4	38.4 ± 4.9	37.1 ± 14.8	31.0 ± 9.2
Male *n* (%)	11 (44)	13 (35)	11 (55)	12 (46.1)	15 (50)
Hydroxyurea *n* (%)		31 (83.7)^a^**	16 (80)^b^**	6 (23)	20 (66.6)^c^*
Osteonecrosis *n* (%)		8 (21.6)	9 (45)^b^*	4 (15.3)	3 (10)
Retinopathy *n* (%)		2 (5.4)^a^*	6 (30)	8 (30.7)	11 (36.6)
Vasculopathy *n* (%)		6 (16.2)	0 (0)	2 (7.7)	5 (16.6)
ACS *n* (%)		16 (43.2)	7 (35)^b^*	2 (7.7)	8 (26.7)
Cholecystectomy *n* (%)		8 (21.6)	10 (50)	5 (19.2)	6 (20)
Splenectomy *n* (%)		0 (0)	1 (5)	2 (7.7)	4 (13.3)
PE/VTE *n* (%)		5 (13.5)	3 (0.15)	1 (3.8)	0 (0)
**Coagulation tests**					
PT (sec)		14.2 [13.2–15.6]	14.3 [13.1–15.5]	14.6 [13.4–15.2]	15.1 [14.5–15.7]
aPTT (sec)		34.3 [32.9–38.1]	35.3 [31.1–39.1]	35.1 [31.9–36.7]	34.5 [33.2–36.4]
Fibrinogen (g/L)		2.75 [2.26–3.36]	2.90 [2.10–3.20]	2.56 [2.24–2.83]	3.09 [2.74–3.52]^c^*
**Hematological parameters**					
RBCs (T/L)		2.85 [2.35–3.42]^a^**	3.04 [2.83–3.57]^b^*	4.02 [3.78–4.64]	2.79 [2.42–3.61]^c^**
Hemoglobin (g/dL)		8.5 [7.8–10.2]^a^*	8.5 [7.9–9.2]^b^**	10.5 [10.1–11.3]	8.8 [7.9–10.2]^c^*
Hematocrit (%)		0.24 [0.22–0.29]^a^*	0.25 [0.23–0.28]^b^*	0.30 [0.29–0.32]	0.25 [0.23–0.29]^c^*
MCV (fL)		88.4 [79.5–105.3]^a^**	83.4 [76.1–86.6]^b^*	73.5 [68.6–78.6]	90.5 [79.8–99.7]^c^**
MCHC (g/dL)		34.9 [32.7–35.8]	33.4 [32.7–33.9]^b^*	35.9 [33.7–35.9]	34.9 [33.9–35.8]^d^*
Platelets (G/L)		365 [244–439]^a^**	326 [216–390]^b^*	186 [159–261]	307 [247–410]
Leukocytes (G/L)		8.3 [7.1–10.2]^a^*	7.0 [5.0–9.4]	6.0 [4.6–6.4]	10.3 [7.7–12.1]^c^**
Neutrophils (G/L)		4.89 [2.98–6.28]	3.86 [2.76–5.04]	3.10 [2.63–3.60]	5.59 [3.99–7.31]^c^*
Lymphocytes (G/L)		2.67 [1.77–3.40]	1.84 [1.34–2.79]	1.76 [1.31–2.70]	2.57 [1.93–3.58]^c^*
Monocytes (G/L)		0.92 [0.57–1.19]^a^**	0.69 [0.53–0.94]	0.45 [0.32–0.62]	0.88 [0.54–1.29]^c^**
**Hemolysis parameters**					
Reticulocytes (G/L)		251.7 [155.5–322.7]^a^**	250.1 [191.1–295.6]^b^*	138.0 [108.7–150.1]	275.9 [180.8–384.9]^c^**
LDH (IU/L)		410 [364–517]^a^**	385 [282–578]^b^*	225 [185–294]	418 [354–497]^c^**
Indirect bilirubin (μmol/L)		25.0 [19.0–37.0]	23.0 [18.0–35.0]	18.0 [15.0–26.0]	29.0 [19.5–45.5]
**Hemoglobin profile**					
HbS (%)		83.6 [65.3–91.05]^a^*	87.2 [62–91.38]^b^*	47.7 [46.6–52.7]	81.9 [48.6–87.5]

Data are expressed as median ± [IQR] except for age (mean ± SD) and clinical characteristics, *n* is the total number of patients (%).

ACS, acute chest syndrome; PE/VTE, pulmonary embolism/venous thromboembolism; PT, prothrombin time; aPTT, activated partial thromboplastin time; RBC, red blood cells; MCV, mean corpuscular volume, MCHC, mean corpuscular hemoglobin concentration; LDH, lactate deshydrogenase; HbS, hemoglobin S.

^a^Indicates a significant difference between S/S-S/β^0^ and S/C-S/β^+^.

^b^Indicates a significant difference between S/Sα^3.7^ and S/C-S/β^+^.

^c^Indicates a significant difference between VOC and S/C-S/β^+^.

^d^Indicates a significant difference between S/Sα^3.7^ and VOC.

*p < 0.05.

**p < 0.001.

### Thrombin generation assay

We evaluated the association between TGA parameters, hemolysis markers (hemoglobin, RET count, LDH, indirect bilirubin), fibrinogen and blood counts in SCD at steady state (Table 2).

**TABLE 2:** Correlation of TGA parameters in all SCD genotypes at steady state.

	Lagtime r (*p-*value)	ETP r (*p*-value)	Peak r (*p*-value)	Time to peak r (*p*-value)	Velocity r (*p*-value)
Fibrinogen (g/L)	0.12 (0.28)	**0.31 (0.003)**	**0.33 (0.002)**	−0.03(0.81)	**0.3 (0.006)**
Leukocytes (G/L)	−0.04(0.70)	0.12 (0.26)	**0.22 (0.04)**	−0.13(0.24)	**0.22 (0.03)**
Monocytes (G/L)	−0.17(0.10)	0.08 (0.44)	**0.32 (0.002)**	−**0.32 (0.003)**	**0.36 (0.0007)**
Reticulocytes (G/L)	−0.09(0.39)	0.21 (0.07)	**0.32 (0.006)**	−0.15(0.19)	**0.30 (0.0089)**
Platelets (G/L)	−**0.32 (0.002)**	0.06 (0.59)	0.03 (0.76)	−**0.39 (0.002)**	0.01 (0.88)
LDH (IU/L)	−**0.27 (0.03)**	0.06 (0.66)	**0.36 (0.005)**	0.35 (0.04)	**0.4 (0.002)**

Data are expressed as r Pearson correlation and (*p*-value). TGA, thrombin generation assay; ETP, endogenous thrombin potential. Bold values are for significant values.

At steady state, we observed a correlation between peak and velocity, in particular, and fibrinogen, leukocytes, monocytes, platelets, RET and LDH. TGA association in the 4 subgroups (S/S-S/β^0^, S/Sα^3^.^7^, S/C-S/β^+^, and VOC) is shown in Supplementary Tables 1–4.

TGA revealed hypercoagulability in patients compared to controls. Lagtime and TTP were significantly lower and associated with increased peak and velocity in all 4 SCD subgroups compared to controls (Table 3). No differences were observed in ETP in S/S-S/β^0^ (*p* = 0.96), S/Sα^3^.^7^ (*p* = 0.27), S/C-S/β^+^ (*p* = 0.45), and VOC (*p* = 0.73). No differences were observed in TGA parameters between S/S-S/β^0^, S/Sα^3^.^7^, S/C-S/β^+^, and VOC.

**TABLE 3:** Comparison of TGA parameters between patients and controls.

TGA parameters	Controls (*n* = 25)	S/S-S/β ^0^ (*n* = 37)	S/Sα ^3.7^ (*n* = 20)	S/C-S/β ^+^ (*n* = 26)	VOC (*n* = 30)
Lagtime (min)	7.50 [6.67–7.93]	4.17 [3.75–4.58]^a^**	4.37 [3.96–4.74]^a^**	4.38 [3.96–4.84]^a^**	4.58 [3.90–5.21]^a^**
ETP (nM.min)	1184 [1101–1282]	1190 [1078–1301]	1114 [912.2–1300]	1212 [1090–1422]	1177 [1040–1380]
Peak (nM)	144.9 [119.1–153.0]	239.7 [196.3–278,1]^a^**	208.8 [165.9–253.8]^a^**	213.6 [173.5–266.9]^a^**	225.2 [194.6–266.9]^a^**
Time to peak (min)	12.71 [11.36–13.14]	7.08 [6.46–8.44]^a^**	7.71 [7.13–8.12]^a^**	7.50 [7.08–8.54]^a^**	7.50 [6.67–8.44]^a^**
Velocity (nM.min^–1^)	28.98 [21.24–33.18]	77.12 [57.31–107.3]^a^**	64.57 [45.83–97.25]^a^**	67.32 [49.18–92.08]^a^**	71.42 [59.97–108.6]^a^**

Data are expressed as median ± [IQR], *n* is the total number of patients. TGA, thrombin generation assay; VOC, vaso-occlusive crisis; ETP, endogenous thrombin potential.

^a^Indicates a significant difference with controls. **p < 0.001.

Then, we pooled the three genotype subgroups at steady state (i.e., S/S-S/β^0^, S/Sα^3^.^7^, and S/C-S/β^+^, *n* = 83) for comparison with the VOC subgroup including all genotypes (Table 4). No differences were observed in thrombin generation parameters between steady state and VOC.

**TABLE 4:** Comparison of TGA parameters between steady state and VOC.

TGA parameters	Steady state (*n* = 83)	VOC (*n* = 30)	*P*-value
Lagtime (min)	4.17 [3.96–4.58]	4.58 [3.90–5.21]	0.11
ETP (nM.min)	1175 [1071–1319]	1177 [1040–1380]	0.65
Peak (nM)	221.3 [177.8–269.1]	225.2 [194.6–266.9]	0.66
Time to peak (min)	7.50 [6.67–8.12]	7.50 [6.67–8.43]	0.63
Velocity (nM.min^–1^)	68.9 [50.9– 100.4]	71.4 [59.9–108.6]	0.50

Data are expressed as median ± [IQR], *n* is the total number of patients. TGA, thrombin generation assay; VOC, vaso-occlusive crisis; ETP, endogenous thrombin potential.

### Physiological anticoagulant proteins

**FIGURE 1:**
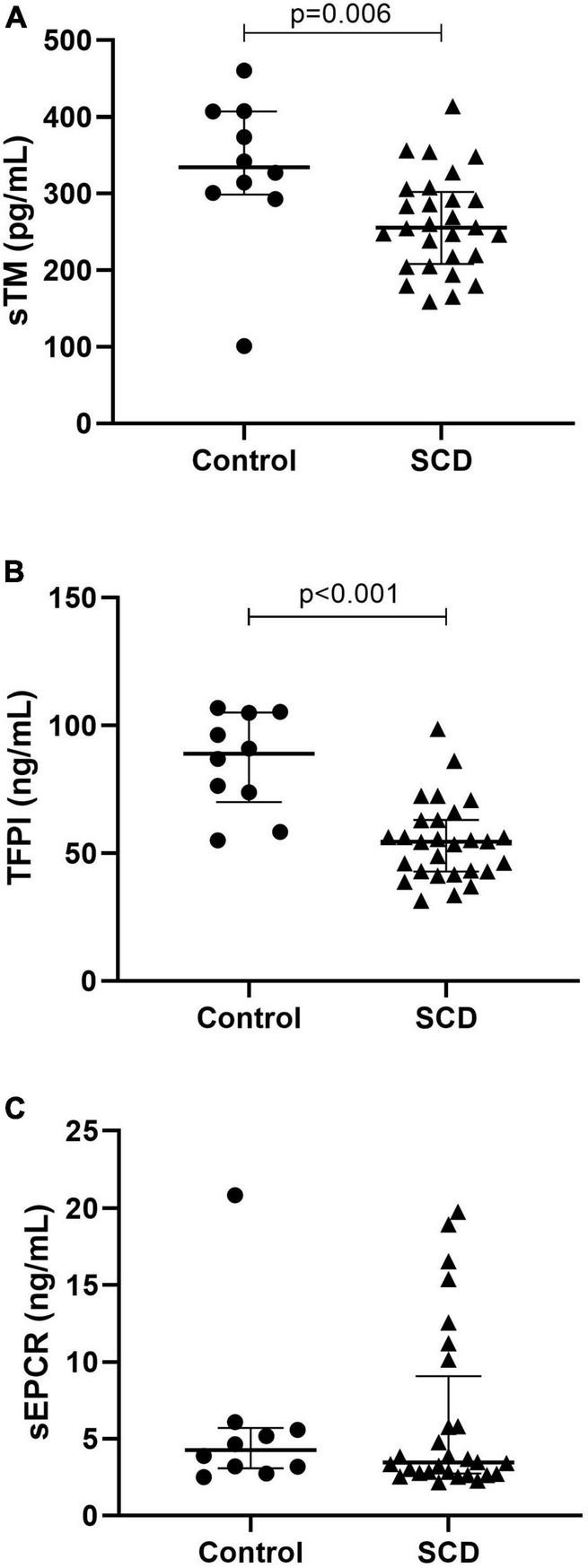
Anticoagulant protein expression in sickle cell disease. With thrombomodulin **(A)**, tissue factor pathway inhibitor **(B)**, and soluble endothelial protein C receptor **(C)**. TFPI, tissue factor pathway inhibitor; sEPCR, soluble endothelial protein C receptor, TM, thrombomodulin; SCD, sickle cell disease. Data are expressed as median [IQR]. *P*-values comparing control group and SCD patients in steady state are from Mann–Whitney test.

We compared TM, TFPI and soluble endothelial protein C receptor (sEPCR) between patients and controls at steady state (Figure 1). Median TM was significantly decreased in patients (255.5 pg/mL, [208.2–302.3]) compared to controls (334.5 pg/mL, [298.9–407.2], *p* = 0.0061). Median TFPI level was significantly decreased in patients (54.54 ng/mL [42.89–63.05]) compared to controls (88.97 ng/mL [69.99–105.1], *p* < 0.0001). Median sEPCR was not different between patients and controls.

We determined differences in TFPI, TM, and sEPCR between the three genotype subgroups at steady state. TFPI was significantly decreased in S/S-S/ß^0^ (46.03 ng/mL, [40.03–59.35]) compared to S/Sα^3^.^7^ (66.12 ng/mL, [58.78–78.47], *p* = 0.046) but not with S/C-S/ß^+^ (54.80 ng/mL [43.18–56.35], *p* = 0.37). TM was decreased in S/S-S/ß^0^ (219.7 pg/mL [179.7–251.0]) compared to S/Sα^3^.^7^ (307.9 pg/mL [264.6–352.2], *p* < 0.001) and S/C-S/ß^+^ (*n* = 10) (288.7 pg/mL [240.4–334.0], *p* = 0.0052). No difference in sEPCR was observed.

### Vaso-occlusive crisis prediction by thrombin generation assay

**FIGURE 2:**
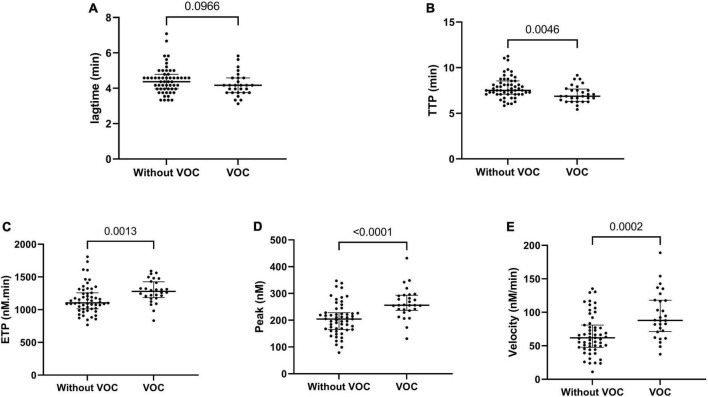
Thrombin generation parameters associated with VOC development in the year following steady state. Lagtime **(A)**, time to peak **(B)**, endogenous thrombin potential **(C)**, peak thrombin **(D)**, and velocity **(E)**. Data are expressed as median [IQR]. P-values comparing SCD in steady state and SCD developing VOC in a year are from Kruskall–Wallis ANOVA with Dunn’s multiple comparisons post-test.

**FIGURE 3:**
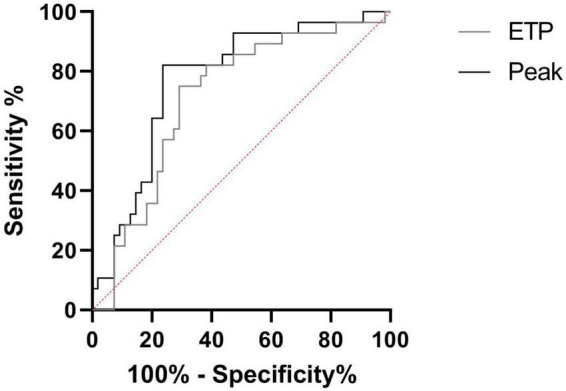
Receiver operating characteristic (ROC) curve of VOC prediction by TGA. Comparison between SCD in steady state and SCD developing VOC in a year. ETP, endogenous thrombin potential (with an ETP > 1,207 nM.min: AUC: 0.71, sensitivity: 75.1%, specificity: 70.9%; With a Peak >228.5 nM: AUC: 0.77, sensitivity: 82.1%, specificity: 76.4%).

We prospectively monitored patients at steady state to determine which of them would be hospitalized for VOC (*n* = 28) within 1 year. Patients with VOC development had decreased TTP and increased ETP, peak and velocity at steady state, compared to patients without VOC development (Figure 2). The risk to develop VOC, determined with a ROC curve, was an ETP of >1,207 nM.min and a peak of >228.5 nM (AUC: 0.71, sensitivity: 75.1%, specificity: 70.9%; AUC: 0.77, sensitivity: 82.1%, specificity: 76.4%, respectively) (Figure 3).

**FIGURE 4:**
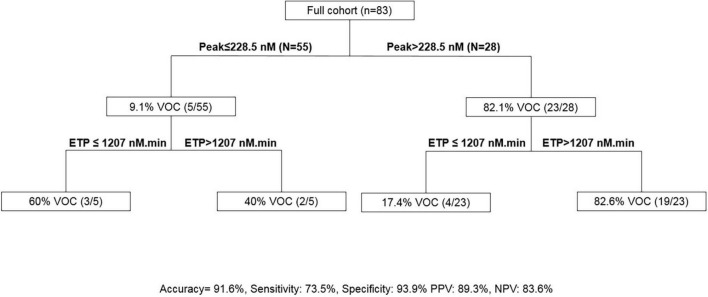
Calibrated automated thrombography (CAT)-based algorithm to predict VOC. ETP, endogenous thrombin potential; PPV, positive predictive value; NPV, negative predictive value, VOC, vaso-occlusive crisis.

The association of an ETP of >1,207 nM.min and a peak of >228.5 nM presented a sensitivity of 73.5% and a specificity of 93.9% to predict VOC in SCD (Figure 4).

## Discussion

Our study aimed to evaluate the use of TGA to assess hypercoagulable state in SCD, and to investigate whether TGA parameters could be used to predict VOC development within 1 year. Our results demonstrate a hypercoagulable state in SCD and a good correlation between ETP-peak and VOC risk of hospitalization within 1 year.

Sickle cell disease has been described as inherited thrombophilia caused by a complex pathophysiology, in particular, chronic hemolysis. VOC are characterized by hemolytic anemia, endothelial damage, and potentially life-threatening complications (26). In addition to hemolysis, acquired hypercoagulability induced several hemostatic changes like TF and phospholipid overexpression, endothelial dysfunction and anticoagulant pathways, with decreased protein S and protein C (5).

Venous thromboembolism is common in adults with SCD and was found in 18.8% of SCD patients (27). We demonstrated a hypercoagulable state on TGA with a significantly shortened lagtime and TTP associated with a higher peak and velocity in all genotypes at steady state and during VOC. Surprisingly, ETP was similar in all four SCD subgroups and the control group. TGA parameters were poorly correlated with hemolysis markers (RETs and LDH) and with leukocytes and monocytes, confirming a complex pathophysiology and a thromboinflammatory hypothesis. However, we were not able to demonstrate a difference between patients at steady state or during VOC. Anticoagulant pathways were previously described with decreased antithrombin, protein S and protein C (4, 28). We demonstrated significantly decreased plasma levels of total TFPI and TM between patients compared to controls. Based on these results, we hypothesize that anticoagulant pathways are chronically consumed in SCD caused by vascular hemolysis and limited ETP (29). Moreover, we observed a non-significant increase of SCD patients with a history of thromboembolism. Our non-significant results are probably secondary to the small number of events.

Several studies have evaluated TGA in SCD. TGA was studied for the first time by Betal et al. in 23 patients with S/S-S/β^0^. These authors reported a significantly lower lagtime, ETP and TTP with no difference in peak (29). Conversely, Shah et al., with the same TGA protocol, reported a significant increase of ETP (*p* < 0.01), peak (*p* < 0.01), and D-dimer (*p* < 0.05) during crisis associated with a lower lagtime (*p* < 0.01) and velocity (*p* < 0.01) in paired adults (21). Gerotziafas et *al* demonstrated no difference in ETP and lagtime associated with a higher peak and velocity and a lower TTP in 92 SCD patients at steady state treated or not with hydroxyurea using 5 pM of TF and 4 μM of PL concentrations (22). Moreover, previous studies demonstrated a lower ETP compared to healthy controls, in SCD patients treated with hydroxyurea and exchange blood transfusion (23, 30). A lack of standardization in performing the assays contributed largely to a poor correlation between assays and study results with TGA (31).

Several studies have demonstrated thrombin generation modification in SCD treated with hydroxyurea (22, 23). Hydroxyurea reduced hypercoagulability in treated patients with a higher lagtime and TTP, a lower peak and velocity and no difference in ETP compared to untreated patients. Only one study revealed no difference between patients treated or not with hydroxyurea with TGA (30). In our study, we did not compare patients treated or not with hydroxyurea because a significant proportion of homozygous patients were treated long-term. Six heterozygous SCD patients were treated according to recommendations for this therapy in patients who reported vasculopathy, ACS or more that 3 VOCs per year (32).

The ability to predict the phenotype of an individual with SCD could guide therapeutic decision making. An interesting result of this study was the use of TGA to predict VOC. Increased ETP and peak were associated with VOC development requiring hospitalization within a year. Moreover, the association of an ETP of >1,207 nM.min and a peak of >228.5 nM presented a sensitivity of 73.5% and a specificity of 93.9% to predict VOC development during the year following the visit. Other scores to predict VOC severity in ACS are emerging (33). New therapeutics are used in SCD to prevent VOC like crizanlizumab, a monoclonal antibody targeted to P selectin (34). Our TGA results could be used to study therapeutics with the objective of preventing VOC.

Anti-platelet and anticoagulant therapies have been investigated in SCD with promising results in pre-clinical studies, but these results were not confirmed in clinical trials (2, 4, 14, 28, 35). In fact, adults with SCD are prone to develop hemorrhagic stroke (2). Recently, contact pathway inhibition was evaluated in mice and may provide a target to reduce hypercoagulable state in SCD (36).

The main limitation of our study is the sample size, with subgroups based on genotype or clinical phenotype. However, these preliminary findings require further exploration in a larger cohort comparing steady state and VOC in paired patients. A second limitation is the diversity of reagents or conditions (PPP, PRP, whole blood) for TGA in current clinical practice which may lead to different results. Moreover, we did not use an inhibitor of contact phase [corn trypsin inhibitor (CTI)], which may have an impact on TG parameters when using 1 pM TF (37). A comparison between PPP with and without CTI or TM would have been interesting to complete our TGA results. Recently, studies showed that TGA with TM was more sensitive to evaluate endothelial dysfunction (17, 18, 38). However, we followed ISTH recommendations to limit the impact (16, 25). Moreover, a new generation of CAT (ST Genesia) could be more standardized and facilitate VOC prediction (38). Finally, we did not explore all endogenous inhibitors of coagulation because lower AT, protein S and C were described. Betal and al hypothesized an upregulation of TFPI in SCD. Here we provide new data on total TFPI, TM, and sEPCR.

## Conclusion

In this study, we have demonstrated, in all SCD genotypes, that a hypercoagulable state is associated with chronic hemolysis. Based on these preliminary findings, ETP and peak could be used to predict VOC development within 1 year.

## Data availability statement

The raw data supporting the conclusions of this article will be made available by the authors, without undue reservation.

## Ethics statement

The studies involving human participants were reviewed and approved by the institutional review board (Rouen University Hospital) approved the study (Authorization protocol number: E2021-78). Written informed consent for participation was not required for this study in accordance with the national legislation and the institutional requirements.

## Author contributions

GF and FK performed the analysis and wrote the manuscript. MG, YB, AL, and CD included patients and critically revised the manuscript and results. VB critically revised the manuscript and results. VL discussed the obtained results and critically revised the manuscript. PB designed the research, analyzed, interpreted the data, and wrote the manuscript. All authors have read and approved the final version of the manuscript.
