# Supplementary material for: Investigation of thrombin generation assay to predict vaso-occlusive crisis in adulthood with sickle cell disease
Source: Front Cardiovasc Med. 2022 Oct 5;9:883812. doi: 10.3389/fcvm.2022.883812 (PMC9579298; doi:10.3389/fcvm.2022.883812)
Supplement: Supplementary file 1 [file Table_1.DOCX]

**Supplemental data**

|  | **Lagtime**  **r (p value)** | **ETP**  **r (p value)** | **Peak**  **r (p value)** | **Time to peak**  **r (p value)** | **Velocity**  **r (p value)** |
| --- | --- | --- | --- | --- | --- |
| RBCs (T/L) | **0.35 (0.02)** | -0.05 (0.74) | -0.26 (0.11) | **0.33 (0.04)** | -0.27 (0.09) |
| Hematocrit (%) | 0.09 (0.59) | -0.14 (0.37) | **-0.34 (0.03)** | 0.3 (0.06) | **-0.35 (0.03)** |
| Monocytes (G/L) | 0.11 (0.51) | 0.09 (0.56) | **0.38 (0.01)** | -0.25 (0.13) | **0.44 (0.006)** |
| Reticulocytes (G/L) | 0.08 (0.65) | **0.4 (0.02)** | 0.3 (0.08) | 0.01 (0.94) | 0.25 (0.16) |
| LDH (IU/L) | -0.28 (0.2) | 0.26 (0.24) | **0.47 (0.03)** | -0.4 (0.06) | **0.51 (0.01)** |

**Supplemental Table 1. Correlation in S/S-S/β^0^.** Data are expressed as r Pearson correlation and (p value)

|  | **Lagtime**  **r (p value)** | **ETP**  **r (p value)** | **Peak**  **r (p value)** | **Time to peak**  **r (p value)** | **Velocity**  **r (p value)** |
| --- | --- | --- | --- | --- | --- |
| Fibrinogen (g/L) | 0.04 (0.83) | **0.45 (0.04)** | 0.32 (0.15) | -0.02 (0.92) | 0.21 (0.36) |
| RBCs (T/L) | -0.3 (0.19) | 0.19 (0.4) | 0.39 (0.08) | **-0.52 (0.01)** | **0.45 (0.04)** |
| Hemoglobin (g/dL) | -0.31 (0.17) | 0.37 (0.1) | **0.49 (0.02)** | **-0.46 (0.04)** | **0.52 (0.01)** |
| Hematocrit (%) | -0.19 (0.41) | 0.35 (0.1) | 0.43 (0.05) | -0.38 (0.09) | **0.44 (0.04)** |
| Platelets (G/L) | **-0.5 (0.02)** | 0.31 (0.18) | 0.32 (0.16) | **-0.5 (0.02)** | 0.29 (0.2) |
| Monocytes (G/L) | **-0.63 (0.003)** | 0.15 (0.5) | 0.17 (0.45) | **-0.54 (0.01)** | 0.14 (0.53) |
| Reticulocytes (G/L) | **-0.51 (0.04)** | 0.49 (0.05) | **0.62 (0.01)** | **-0.66 (0.004)** | **0.63 (0.009)** |
| LDH (IU/L) | -0.37 (0.16) | 0.33 (0.22) | 0.46 (0.08) | **-0.54 (0.03)** | 0.45 (0.08) |

**Supplemental table 2. Correlation in S/Sα^3.7^.** Data are expressed as r Pearson correlation and (p value)

|  | **Lagtime**  **r (p value)** | **ETP**  **r (p value)** | **Peak**  **r (p value)** | **Time to peak**  **r (p value)** | **Velocity**  **r (p value)** |
| --- | --- | --- | --- | --- | --- |
| Hemoglobin (g/dL) | -0.16 (0.42) | **0.4 (0.04)** | **0.39 (0.04)** | -0.23 (0.24) | 0.36 (0.06) |
| Leukocytes (G/L) | -0.06 (0.76) | **0.46 (0.01)** | **0.45 (0.02)** | -0.12 (0.53) | **0.43 (0.02)** |
| Lymphocytes (G/L) | -0.18 (0.35) | 0.38 (0.05) | **0.42 (0.03)** | -0.21 (0.28) | 0.38 (0.05) |
| Monocytes (G/L) | -0.33 (0.09) | 0.33 (0.09) | **0.49 (0.01)** | -0.37 (0.05) | **0.52 (0.005)** |
| Fibrinogen (g/L) | 0.13 (0.54) | **0.49 (0.01)** | **0.44 (0.02)** | -0.03 (0.84) | **0.39 (0.04)** |
| HbS (%) | 0.13 (0.54) | -0.31 (0.13) | **-0.43 (0.03)** | 0.36 (0.08) | -0.37 (0.06) |

**Supplemental table 3. Correlation in S/C-S/β^+^**.Data are expressed as r Pearson correlation and (p value)

|  | **Lagtime**  **r (p value)** | **ETP**  **r (p value)** | **Peak**  **r (p value)** | **Time to peak**  **r (p value)** | **Velocity**  **r (p value)** |
| --- | --- | --- | --- | --- | --- |
| Fibrinogen (g/L) | **0.42 (0.02)** | 0.18 (0.31) | 0.15 (0.43) | 0.23 (0.2) | 0.2 (0.28) |
| Platelets (G/L) | **-0.39 (0.03)** | -0.01 (0.92) | 0.28 (0.12) | -0.35 (0.05) | 0.27 (0.14) |
| Leucocytes (G/L) | -0.26 (0.15) | -0.31 (0.09**)** | 0.27 (0.14) | **-0.37 (0.04)** | 0.3 (0.1) |
| Neutrophils (G/L) | -0.2 (0.26) | -0.21 (0.26) | 0.35 (0.05) | -0.35 (0.05) | **0.37 (0.04)** |
| Lymphocytes (G/L) | -0.19 (0.31) | **-0.49 (0.006)** | -0.11 (0.56) | -0.18 (0.33) | -0.03 (0.85) |

**Supplemental table 4. Correlation in VOC.** Data are expressed as r Pearson correlation and (p value)
